# Two-stage approach for identifying single-nucleotide polymorphisms associated with rheumatoid arthritis using random forests and Bayesian networks

**DOI:** 10.1186/1753-6561-1-s1-s56

**Published:** 2007-12-18

**Authors:** Yan Meng, Qiong Yang, Karen T Cuenco, L Adrienne Cupples, Anita L DeStefano, Kathryn L Lunetta

**Affiliations:** 1 Genetics Program, Department of Medicine, School of Medicine, Boston University, 715 Albany Street, Boston, Massachusetts 02118 USA; 2 Department of Biostatistics, School of Public Health, Boston University, 715 Albany Street, Boston, Massachusetts 02118 USA; 3 Department of Neurology, School of Medicine, Boston University, 715 Albany Street, Boston, Massachusetts 02118 USA; 4 Current affiliation: Center for Human Genetic Research, Massachusetts General Hospital, Boston, Massachusetts 02114 USA; Broad Institute of Harvard and Massachusetts Institute of Technology, Cambridge, Massachusetts 02139 USA.

## Abstract

We used the simulated data set from Genetic Analysis Workshop 15 Problem 3 to assess a two-stage approach for identifying single-nucleotide polymorphisms (SNPs) associated with rheumatoid arthritis (RA). In the first stage, we used random forests (RF) to screen large amounts of genetic data using the variable importance measure, which takes into account SNP interaction effects as well as main effects without requiring model specification. We used the simulated 9187 SNPs mimicking a 10 K SNP chip, along with covariates DR (the simulated DRB1 gentoype), smoking, and sex as input to the RF analyses with a training set consisting of 750 unrelated RA cases and 750 controls. We used an iterative RF screening procedure to identify a smaller set of variables for further analysis. In the second stage, we used the software program CaMML for producing Bayesian networks, and developed complex etiologic models for RA risk using the variables identified by our RF screening procedure. We evaluated the performance of this method using independent test data sets for up to 100 replicates.

## Background

It is commonly believed that complex diseases are caused not by single genes acting alone, but by multiple genes and non-genetic factors interacting with one another. Due to the large number of single-nucleotide polymorphisms (SNPs) now available in genome-wide scans, the computational burden of testing each locus for main effects and all possible two-way, three-way, and higher-order interactions is overwhelming. One approach to reducing the number of interactions to examine is to perform a two-stage analysis. In the first stage, one identifies a subset of SNPs for further analysis of interaction in the second stage. Often, a univariate test (e.g., a chi-square test) is used to identify SNPs associated with outcome in the first stage. When risk-associated SNPs have small marginal effects but large interaction effects in the population, univariate methods will result in low power for detecting these SNPs. "Multi-locus" approaches consider interactions of multiple genes and environmental factors in identifying susceptibility loci for complex diseases [1]. Random Forests (RFs) [2] provide a powerful method for detecting interacting risk susceptibility SNPs (rSNPs) [3]. However, this method does not provide a model that delineates the interactions.

Bayesian networks (or directed graphical models) are graphs in which the nodes represent random variables and the arrows represent dependence relationships [4]. These methods have been successfully applied to generate a model describing the relationship among SNPs in multiple candidate genes for a complex trait [5].

## Methods

We used the 100 replicates of simulated data in Problem 3 provided by the Genetic Analysis Workshop 15 (GAW15). We performed analyses with knowledge of the "real" answers but screened all of the 9187 SNPs, distributed on the genome to mimic a 10 K SNP chip without regard to the generating model. We used disease status for rheumatoid arthritis (RA) as the outcome and smoking, sex and DR genotype (the simulated DRB1 genotype) as covariates.

### Subjects

To obtain biologically independent cases, for each replicate we randomly selected one affected sibling from each of 1500 nuclear families. These 1500 cases were then divided at random into a training data set of 750 affected subjects and a test data set of 750 cases. The GAW data provided 2000 unrelated unaffected individuals for use as controls. Two independent sets of 750 controls were selected at random from the 2000 for use as training data set and test data set controls. Thus, for each replicate we had independent training and test data sets consisting of 750 cases and 750 controls.

### Random Forests

RFs grow a large number of classification or regression trees with no trimming or pruning. The RF method produces an importance score for each variable that quantifies the relative contribution of that variable to the prediction accuracy. We used this score to prioritize the predictor variables. The RF also produces prediction errors for the individuals, which we used for evaluation of the method.

We used Random Forests version 5 [6] to analyze the training data. We used an iterative process similar to a strategy previously proposed for gene expression analysis [7] in which, at each iteration, we built a random forest using the training data, and saved the 50% of variables with the highest importance scores to build the next forest. The random forests built at each iteration were named IT_0_, IT_1_,..., IT_*n*_, and the prediction errors of the training data set were estimated for the forest built at each iteration. We call the forest with the best prediction error IT_*bp*_. The variables included in IT_*bp *_were then used in second-stage analysis. We compared the performance of the iterative procedure that resulted in the forest IT_*bp*_, in terms of keeping the true risk variables and removing noise variables, to a simple procedure of selecting the top 50 ranked variables by importance from iteration 0, in the test data sets. Specifically, we compared the prediction error of the IT_*bp *_forest, the IT_0 _forest (all variables used; no selection), and a forest built using only the top 50 SNPs from the IT_0 _forest ("IT_top50_"). Because the iterative procedure averaged 53 SNPs in the final forest, we chose 50 SNPs from IT_0 _to yield a forest with approximately the same number of SNPs. We computed prediction error using the test data ("test"), and using the out-of-bag observations of the training data set ("training").

### Network inference

**Figure 1:**
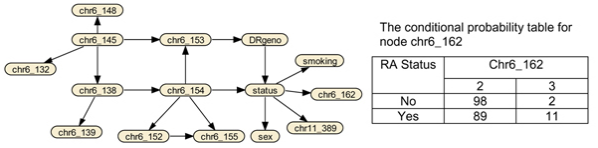
Bayesian network based on variables of IT_bp _for Replicate 1.

Bayesian networks (BN) are directed acyclic graphs for representing the joint probability distribution of all variables. A network for discrete variables, e.g., Figure 1, is specified by the graph structure (nodes and arcs) and the conditional probability table (CPT) at each node (node chr6_162 is shown). Each node is a variable, and each directed arc implies association and direction of dependency between the two variables. The origin node of an arc is usually called the parental node, and nodes that an arc points to are called child nodes. A child node is conditionally independent of other nodes given its parental nodes. Thus, the joint probability of *n *variables can be simplified to $P(x_{1},x_{2},...,x_{n})=\prod_{i=1}^{n}P(x_{i}|x_{i1},x_{i2},...,x_{ik_{i}})$, where *x*_*i*l_, *x*_*i*2_,..., *x*_*ik *_in the condition are the parental nodes of *x*_*i *_and a subset of *x*_1_, *x*_2_, *x*_*i*-1_,..., *x*_*i*+1_, *x*_*n*_. BN models are useful for describing complex relationships among variables, as well as for making predictions for variables that are regarded as outcomes.

CaMML [8], Causal Minimum Message Length (MML), is a program for generating Bayesian networks. The general goal is to find a model that maximizes the posterior probability of that model given the data. CaMML searches over all possible structures (models) using the Metropolis algorithm. It uses MML as a metric that includes a penalty on model complexity to control the resampling process. We evaluated the performance of CaMML on a set of variables used in the IT_*bp *_forest described above. We used the test data set to predict case status and estimate prediction error.

## Results

We identified the best surrogates for all risk loci (A-G) as the SNPs with the highest linkage disequilibrium (LD) (*r*^2^) with risk loci from the answer files given with the GAW15 data (Table 1). For locus C, three SNPs had *r*^2 ^≥ 0.2; for locus D, two SNPs had *r*^2 ^≥ 0.2. When analyzing the results, we considered these SNPs true positives, in that they are the best proxies for the true risk loci that were not genotyped.

**Table 1:** Power estimate of IT_bp _and CaMML

Variables	Disease locus	*R*^2 ^with disease locus	IT_bp _100 replicates	IT_bp _Replicate 1–50	CaMML Replicate 1–50
**DR genotype**^a^	NA	NA	**100%**	**100%**	**100%**
**Sex**	NA	NA	**100%**	**100%**	**100%**
**Smoking**	NA	NA	**96%**	**96%**	**96%**
**chr 6_154**	**C**	**0.958**	**100%**	**100%**	**100%**
**chr 6_153**	**C**	**0.563**	**100%**	**100%**	**100%**
**chr 6_152**	**C**	**0.418**	**100%**	**100%**	**100%**
chr 6_155	C	0.104	97%	98%	98%
chr 6_150	C	0.027	13%	8%	4%
chr 6_149	C	0.014	6%	8%	4%
chr 6_139	C	0.009	18%	20%	10%
chr 6_138	C	0.009	17%	18%	10%
chr 6_140	C	0.007	1%	2%	2%
chr 6_134	C	0.007	4%	4%	4%
chr 6_137	C	0.006	2%	2%	2%
chr 6_130	C	0.006	8%	6%	2%
chr 6_148	C	0.005	9%	8%	4%
chr 6_147	C	0.004	9%	10%	8%
chr 6_135	C	0.002	3%	6%	2%
chr 6_145	C	0.001	35%	32%	24%
chr 6_132	C	0.0	7%	6%	6%
**chr 6_162**	**D**	**0.902**	**100%**	**100%**	**100%**
**chr 6_160**	**D**	**0.273**	**67%**	**68%**	**66%**
chr 6_156	D	0.001	11%	14%	2%
chr 11_387	F	0.135	5%	6%	6%
chr 11_388	F	0.064	5%	4%	4%
**chr 11_389**	**F**	**0.934**	**98%**	**100%**	**100%**
chr 11_391	F	0.031	1%	2%	2%
chr 16_29	A	0.001	1%	0%	0%
chr 18_269	E	0.171	51%	48%	10%
chr 8_442	B	0.001	0%	0%	0%
chr 9_186	G	0.021	0%	0%	0%
chr 9_189	H	0.014	0%	0%	0%

^a^Surrogates and covariates are in bold.

### Risk variables identified by RF

We compared IT_*bp *_and IT_0 _top 50 for choosing a set of variables by comparing how often the best surrogates for loci A-G appeared in the variable set. DR and the best surrogates for C, D, and F were included in 94 and 98 out of 100 replicates for the IT_*bp *_forest and the top 50 variables for IT_0 _forest, respectively. The average number of variables included in the IT_*bp *_forest was 53 (range 8–287). The IT_*bp *_forest occurred, on average, at iteration 7.64 (range 5–10).

### Estimate of prediction error

As seen in Table 2, the mean and median prediction error for the training data sets is smaller than that for the test data sets for the IT_*bp *_and IT_top50 _methods (median differences -2.77, -0.93, *p *< 0.0001), which may indicate overfitting. The IT_0 _forest gives similar prediction error for test and training data.

**Table 2:** Prediction error for random forest analyses

	IT_bp_		IT_top50_		IT_0_		
				
Statistics	Training	Test	Training	Test	Training	Test	CaMML Test
Mean	11.28	14.05	12.73	13.60	14.60	14.73	12.42
SD	0.83	0.90	0.95	0.85	0.96	0.91	0.97
Min	9.80	12.20	10.93	11.60	12.27	12.20	10.35
Max	14.73	16.87	16.00	15.47	18.00	16.53	16.00
*p*-Value^a^	5.26 × 10^-18^		1.35 × 10^-9^		0.25		
Difference in median	-2.77		-0.93		-0.17		

^a^*p*-Value of the paired Wilcoxon rank test comparing training and test data prediction error.

For the training data sets, the mean prediction error for the IT_*bp *_forests is smaller than that for the IT_0 _forests; the IT_top50 _forests fall in between (Table 3). For the test data sets, although both IT_top50 _and IT_*bp *_outperform IT_0_, the IT_*bp *_has larger prediction error than IT_top50 _(difference in median = 0.43, *p *< 0.0001), which might be due to overfitting for the iterative method.

**Table 3:** Paired Wilcoxon rank test of prediction errors from three RFs, using Replicates 1–100

	Training data		Test data	
		
Comparison of prediction errors	*p*-Value	Difference in median	*p*-Value	Difference in median
IT_bp _vs. IT_top50_	3.94 × 10^-18^	-1.40	9.09 × 10^-10^	0.43
IT_bp _vs. IT_0_	3.95 × 10^-18^	-3.33	2.57 × 10^-12^	-0.73
IT_top50 _vs. IT_0_	3.94 × 10^-18^	-1.87	1.20 × 10^-17^	-2.10

### Network inference

We used CaMML to analyze the variables selected from IT_*bp *_for Replicates 1 to 50. Due to computational limits, if more than 50 variables were selected by IT_*bp*_, only the top 50 variables were used for second-stage analysis. With the maximum number of variables restricted to 50, the average number of variables used in CaMML across the 50 replicates was 40. In the estimated BNs, an average of 11 variables were connected to RA status directly or indirectly through other variables in a path of a network that included RA status. The average prediction error using the test data was 12.4% (Table 2), which is smaller than that of IT_*bp *_(Table 4). An example BN with the conditional probability table (CPT) for node chr6_162, using Replicate 1 is displayed in Figure 1. In this BN, all SNPs included in the analysis with *r*^2 ^> 0.3 with one of the disease loci (Table 1) were connected directly or indirectly to RA. Many SNPs on chromosome 6 were interconnected due to LD between these markers. The CPT for node chr6_162 showed 5.5-fold increased risk of RA for carrying allele 3 versus allele 2.

**Table 4:** Paired Wilcoxon rank test of prediction errors from three RFs and CaMML using test data and Replicates 1–50

	Test data	
	
Comparison of prediction errors	*p*-Value	Difference in median
CaMML vs. IT_bp_	1.10 × 10^-8^	-1.52
CaMML vs. IT_top50_	1.04 × 10^-6^	-1.13
CaMML vs. IT_0_	2.16 × 10^-9^	-2.32

Table 1 displays the frequency of variables appearing in the network for Replicates 1–50. We have 100% power to detect SNP6_152, SNP6_153, SNP6_154 (surrogates for locus C), SNP6_162 (surrogate for locus D), and SNP11_389 (surrogate for locus F), all of which have strong LD (*r*^2 ^≥ 0.418) with disease loci. We have lower power (66%) to detect SNP6_160, a surrogate for D that is in lower LD (*r*^2 ^= 0.273). Despite its low LD with locus C (*r*^2 ^= 0.104), the power to detect SNP6_155 is 98%. This may be due to the very strong effect of locus C. Importantly, CaMML identified all covariates (DR, sex, and smoking) and almost all surrogates in LD with disease loci (with exception of SNP6_160, which was not detected by CaMML in one replicate) as part of the RA network from variables selected from IT_*bp*_.

## Discussion

Using the simulated data from Problem 3, we assessed a two-stage approach for identifying SNPs associated with RA that employs random forests to identify important variables, and Bayesian networks to further filter out noise SNPs by reducing prediction error. The random forest analysis reduced the number of variables for further Bayesian network analyses from 9190 to about 53. This screening strategy successfully filtered out many SNPs unassociated with the disease loci, while keeping the surrogates for risk SNPs for four out of nine of these loci (DR, C, D, and F) in 94 of 100 replicates. Although IT_*bp *_seems to give lower prediction error than IT_top50 _in training data sets, IT_top50 _gives lower prediction error than IT_*bp *_in test data sets. Therefore, the strategy of building a second forest using the top 50 SNPs from a first forest may be a better variable selection method overall. However, the effects of these loci in this data set are very strong, and it is not clear that this result will generalize to data weaker association signals. Further, it is not clear how to choose the number of variables to select if one uses the simpler procedure. Additional simulation studies are needed to determine how to generalize our results to less ideal circumstances. The fact that the difference in the median of prediction errors for training and test data sets are large for IT_*bp *_suggests overfitting; however, because we removed a large (50%) proportion of "noise" in this first stage, IT_*bp *_is not expected to be the optimal RF with the lowest prediction error. It is possible to remove one noise variable at a time; however, it is not practical in the context of thousands of variables. We expected the BN analysis to further reduce the number of noise SNPs and provide some guidance as to important interaction effects.

Bayesian network analysis based on a subset of the variables (≤ 50) selected from IT_*bp *_captured most of the true loci and the correct dependencies among them and further decreased the test set prediction error. The network model provides a method for predicting case status and facilitates the understanding of complex relationships between the disease and genetic and environmental factors. The limitations of BN include the difficulty to discern the exact relationship between variables that are interconnected and the exponential increase in computation time with the number of variables. These make BN impractical for genome-wide scan of dense SNPs. However, BN results are at least useful to generate potentially biological meaningful hypotheses to be confirmed by further statistical analyses or/and biological experiments.

## Competing interests

The author(s) declare that they have no competing interests.

## References

[B1] Hoh J, Ott J (2003). Mathematical multi-locus approaches to localizing complex human trait genes. Nat Rev Genet.

[B2] Breiman L (2001). Random forests. Mach Learn.

[B3] Lunetta KL, Hayward LB, Segal J, Van Eerdewegh P (2004). Screening large-scale association study data: exploiting interactions using random forests. BMC Genet.

[B4] Murphy K A brief introduction to graphical models and Bayesian networks. http://www.cs.ubc.ca/~murphyk/Bayes/bnintro.html.

[B5] Sebastiani P, Ramoni MF, Nolan V, Baldwin CT, Steinberg MH (2005). Genetic dissection and prognostic modeling of overt stroke in sickle cell anemia. Nat Genet.

[B6] Breiman L, Cutler A Random forests. Version 5. http://www.stat.berkeley.edu/users/breiman/RandomForests/.

[B7] Diaz-Uriarte R, Alvarez de Andres S (2006). Gene selection and classification of microarray data using random forest. BMC Bioinformatics.

[B8] Wallace CS, Korb KB, Gammerman A (1999). Learning linear causal models by MML sampling. Causal Models and Intelligent Data Management.

